# Development and Characterization of *Phlomis brachyodon* Extract-Loaded Thermoresponsive In Situ Gels: Antioxidant Activity, Wound-Healing Evaluation in a Rat Excision Model, and Potential Anticancer Activity

**DOI:** 10.3390/gels12100909

**Published:** 2026-10-09

**Authors:** Nour Aboalhaija, Fatimah AL-Dulaimi, Osama H. Abusara, Fatma Afifi, Heba Syaj, Baraa Hajjaj, Wasan Al-Farhan, Rania Hamed

**Affiliations:** 1 Department of Pharmacy, Faculty of Pharmacy, Al-Zaytoonah University of Jordan, Amman 11733, Jordan; n.aboalhaija@zuj.edu.jo (N.A.); fatimaaldlimi@gmail.com (F.A.-D.); o.abusara@zuj.edu.jo (O.H.A.); hebasyaj1997@gmail.com (H.S.); baraaalhjjaj4@gmail.com (B.H.); wasanmortada@gmail.com (W.A.-F.); 2 Department of Pharmaceutical Sciences, Faculty of Pharmacy, The University of Jordan, Amman 11942, Jordan; fatueafi@ju.edu.jo

**Keywords:** antioxidant activity, *Phlomis brachyodon*, thermoresponsive in situ gel, wound healing, LC–MS

## Abstract

This study aimed to characterize butanol and methanol extracts of *Phlomis brachyodon* (*P. brachyodon*) and incorporate them into thermoresponsive in situ gels (F1 and F2, respectively) to evaluate their wound-healing activity. Total phenolic content (TPC), total flavonoid content (TFC), and antioxidant activity of the extracts were determined. Extracts were profiled by LC–MS. The rheological properties of the gels, using a controlled-stress rheometer, and their wound-healing activity and histological examinations were evaluated. Cell viability assays of the extracts were conducted against human gingival fibroblasts (HGF) and a small-cell lung cancer cell line (H69). The methanol extract showed higher TPC and TFC values and stronger antioxidant activity than the butanol extract (IC_50_, 55.82 ± 8.94 versus 144.11 ± 10.95 µg/mL). F1 and F2 underwent a sol–gel transition at temperatures suitable for topical application and exhibited shear-thinning behavior with predominantly elastic behavior. Both extract-loaded gels promoted wound contraction, with F2 producing the greatest reduction in wound area and near-complete closure by day 15. F1 and F2 gels showed complete re-epithelialization. LC–MS profiling detected 37 constituents and showed greater chemical diversity in the methanol extract. Both extracts had selectivity indices greater than 30 against H69 cancer cells, with IC_50_ values of 3.16 and 2.24 µg/mL for the butanol and methanol extracts, respectively. *P. brachyodon* extract-loaded thermoresponsive gels, particularly the methanol extract formulation, showed promising antioxidant and wound-healing activity. In conclusion, these findings support the development of *P. brachyodon* topical formulations and demonstrate anticancer activity, particularly against H69 lung cancer cells, for *P. brachyodon* extracts.

## 1. Introduction

Medicinal plants have long been a fundamental source of therapeutic agents and continue to play a vital role in modern healthcare [1]. This is mainly due to their rich array of bioactive constituents, including alkaloids, terpenoids, phenolics such as flavonoids, and tannins [2]. Globally, more than 80% of the population in over 170 of WHO’s 194 Member States currently use some form of traditional medicine, including herbal medicine, yoga, Ayurveda, acupuncture and acupressure, and indigenous therapies [3].

The genus *Phlomis* (family Lamiaceae) is represented by ten species in Jordan. *P. brachyodon* (Boiss.) Zohary ex Rech. F. (syn.: *P. orientalis* Miller var. *brachyodon* (Boiss.) Boiss.) is one of the representatives of this genus distributed primarily in the Mediterranean and Irano-Turanian biogeographic zones of the country, with a large population size [4,5]. The species is characterized by having few erect stems, either simple or branched, and ovate–oblong to lanceolate leaves measuring 4–10 × 3–5.8 cm, and attractive yellow flowers. The leaves exhibit a thick texture and obscure crenulation [6]. Several *Phlomis* species are widely used in traditional medicines for treating wounds, inflammation, gastrointestinal disorders, and respiratory conditions [7]. They are also reported to possess analgesic activity [8] and untiulcer potential [9]. Experimental studies have confirmed a broad range of biological activities, including antioxidant, antimicrobial, anti-inflammatory, and antimutagenic effects [10].

Thermoresponsive in situ gels have emerged as promising systems for topical drug delivery, offering improved retention, enhanced skin hydration, and controlled drug release. These systems undergo a sol–gel transition at body temperature, allowing localized drug delivery and reduced systemic exposure [11].

Wound healing is a complex process that is often impaired by oxidative stress and microbial infection [12]. Plant-derived compounds, particularly phenolics and flavonoids, play a crucial role in promoting healing by reducing oxidative damage, enhancing collagen synthesis, and supporting tissue regeneration [13,14]. Their combined antioxidant, anti-inflammatory, and antimicrobial effects contribute to improved wound healing outcomes [15]. Moreover, several plant extracts are known to possess anticancer activity, as evidenced by various studies [16,17]. Additionally, a plant from the genus *Phlomis* has been shown to possess anticancer activity against cervical and rhabdomyosarcoma cancer cell lines [18].

This study aimed to integrate the phytochemical characterization of *P. brachyodon* extracts with their incorporation into a thermoresponsive topical delivery system and subsequent biological evaluation in an excision-wound model. To our knowledge, the topical application of *P. brachyodon* extracts using a thermoresponsive in situ gel has not previously been investigated. The thermoresponsive approach may offer practical advantages for topical wound-healing applications, where the formulation can be applied in a relatively fluid state and then form a more structured gel at a temperature close to skin temperature, which may facilitate application and local retention. Additionally, this study provides a proof of concept for the anticancer potential of *P. brachyodon* extracts on the H69 lung cancer cell line and indicates that further formulation optimization, safety evaluation, release studies, anticancer potential on different cancer cell lines, and adequately powered animal studies are needed before clinical translation can be considered.

## 2. Results and Discussion

### 2.1. Determination of Total Phenol Content (TPC), Total Flavonoid Content (TFC), and Antioxidant Activity of P. brachyodon Extracts

The butanol extract contained 94.29 ± 3.92 mg GAE/g and 446.31 ± 24.73 mg RE/g, whereas the methanol extract contained 183.96 ± 8.44 mg GAE/g and 1115.64 ± 42.09 mg RE/g. The methanol extract showed stronger 2,2-diphenyl-1-picrylhydrazyl (DPPH) radical-scavenging activity than the butanol extract, with IC_50_ values of 55.82 ± 8.94 and 144.11 ± 10.95 µg/mL, respectively (Table 1). Ascorbic acid, used as a positive control, had an IC_50_ of 6.96 ± 3.53 µg/mL.

### 2.2. Preparation and Characterization of the Blank In Situ Gels

To determine the sol–gel transition temperature (Tsol → gel), blank in situ gels (G1–G7) were prepared using different concentrations of Pluronic F127 (F127) and Kolliphor P188 (F68), and without *P. brachyodon* extracts. No sol–gel transition was detected for G1. The in situ gels G2, G3, and G5 showed transition temperatures between 37 and 50 °C, higher than the skin surface temperature (32–34 °C) [19], whereas G4 transitioned at a lower temperature of 30.5 °C. Therefore, G1–G5 in situ gels were excluded from further characterization. However, G6 and G7 gels showed transition temperatures of 33.75 ± 0.25 and 32.50 ± 0.50 °C, respectively. Therefore, the blank G6 and G7 gels were selected as the optimal gels to load the butanol and methanol extracts of *P. brachyodon*, resulting in F1 and F2 gels, respectively.

### 2.3. Preparation and Characterization of P. brachyodon Extract-Loaded In Situ Gels

**Figure 1:**
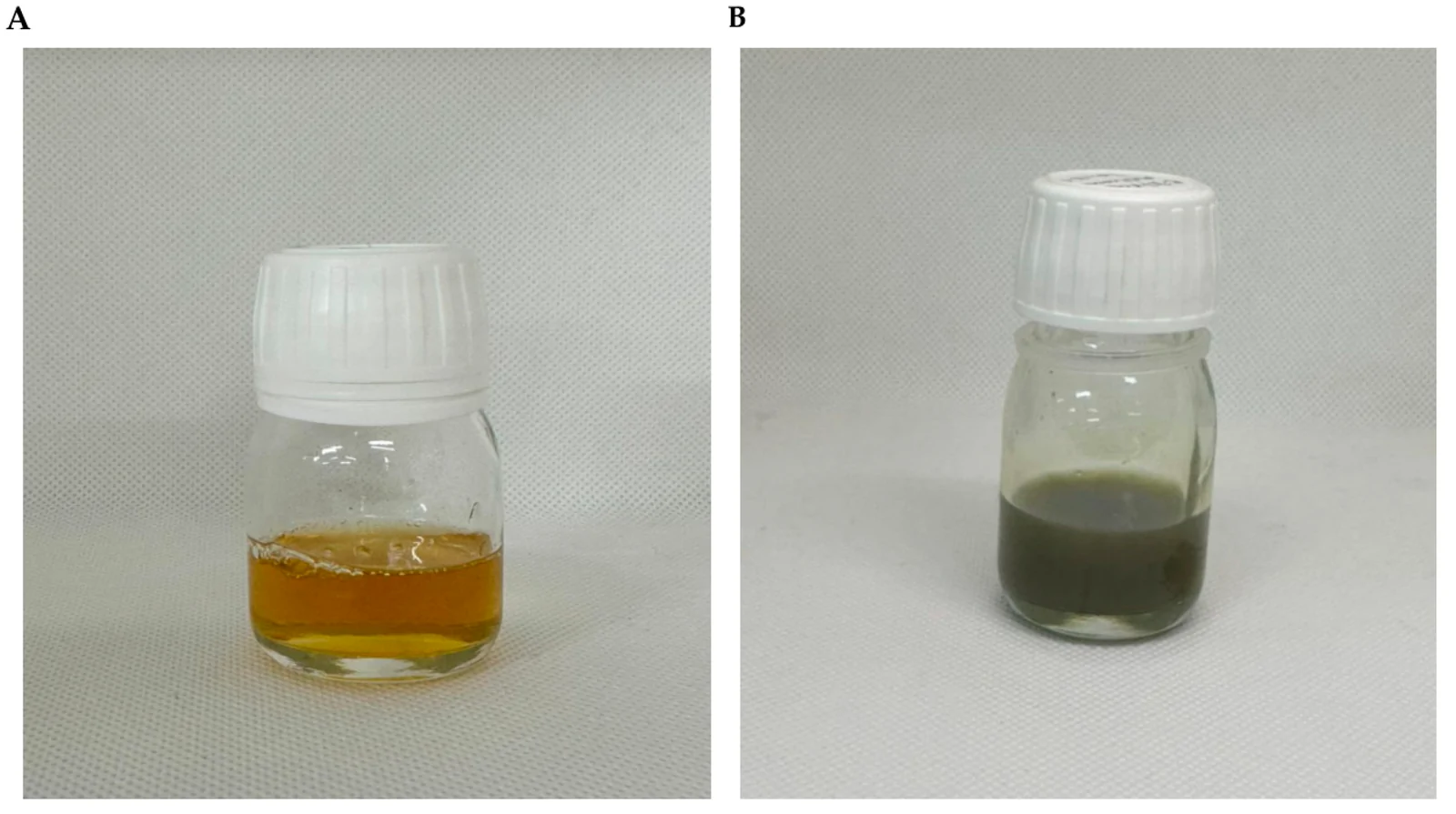
Representative images of (**A**) *P. brachyodon* butanol extract-loaded in situ gel (F1) and (**B**) *P. brachyodon* methanol extract-loaded in situ gel (F2).

The thermoresponsive in situ gels F1 and F2 were prepared using G6 and G7 polymeric bases and loaded with butanol and methanol extracts, respectively (Figure 1). This is because these gels exhibited optimal Tsol → gel temperatures, suitable for skin delivery [20,21]. F1 was dark yellow and clear, whereas F2 was dark green and opaque. Both gels contained F127 and F68, which form a mixed micellar network suitable for incorporating plant extracts [22,23]. By the tube-inversion method, F1 and F2 showed Tsol → gel values of 33.70 ± 0.20 and 31.10 ± 0.45 °C, respectively, with gelation times (Tgel) of 3.70 ± 0.10 and 3.20 ± 0.09 min (Table 2).

### 2.4. Rheological Measurements

#### 2.4.1. Viscosity

**Figure 2:**
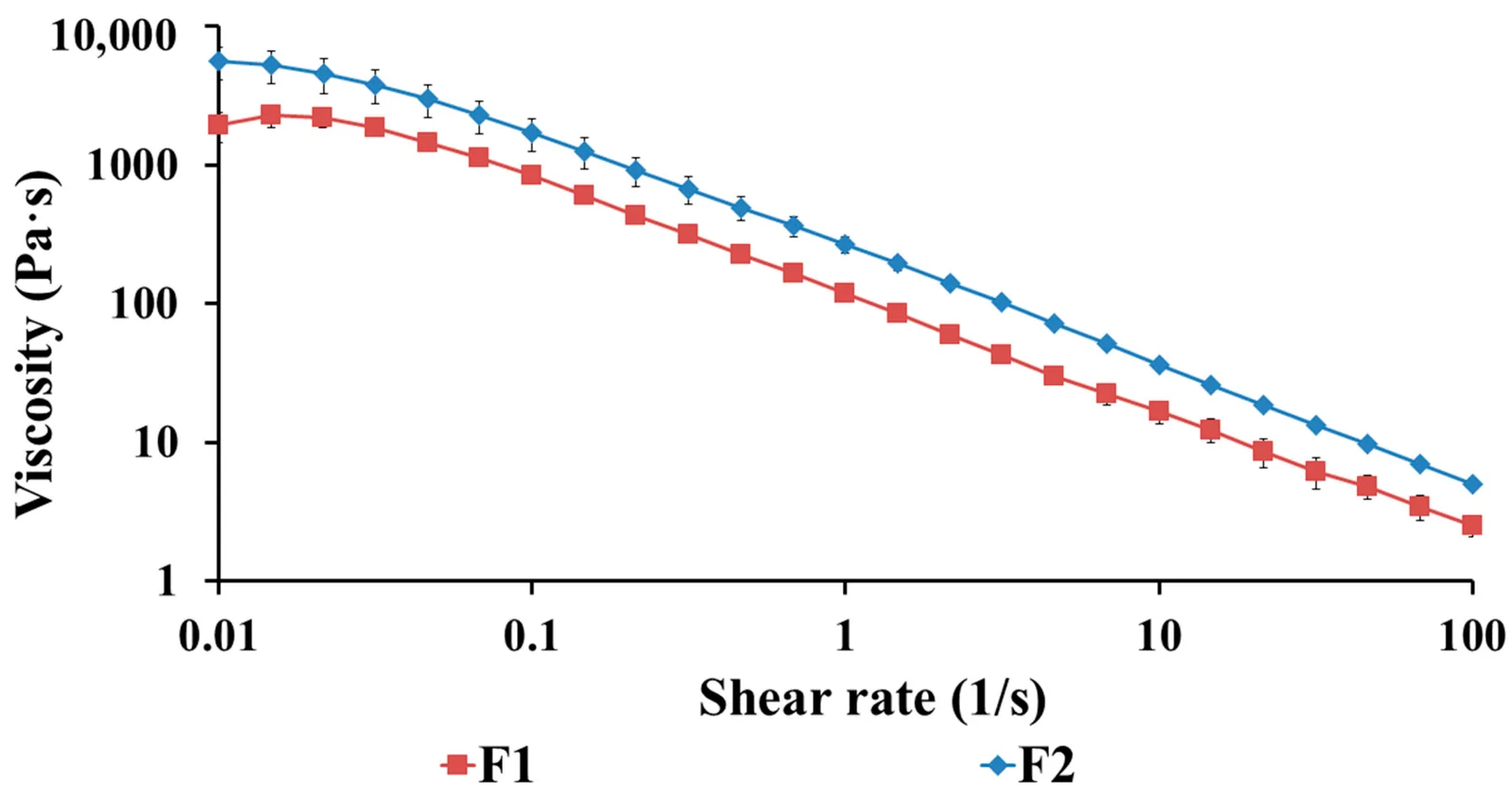
Viscosity curves of F1 and F2 at 32 °C over shear rates of 0.01–100 s^−1^. Data are presented as mean ± SD (n = 3).

Both gels exhibited a shear-thinning behavior (pseudoplastic flow) at 32 °C, with viscosity decreasing as the shear rate increased (Figure 2) [24]. F2 was more viscous than F1; at 0.1 s^−1^, the mean viscosities were 1930 ± 468.9 and 4117 ± 98.6 Pa·s for F1 and F2, respectively. This is because F2 gel contains higher concentrations of F127 and F68 than those of F1, where F2 was composed of 20.8% F127 and 10.0% F68, whereas F1 was composed of 19.0% F127 and 9.0% F68. As a result, gel viscosity decreased with decreasing F127 concentration, consistent with earlier reports [25].

#### 2.4.2. Amplitude-Sweep

The linear viscoelastic region (LVR) of gels was defined by approximately constant storage (G′) and loss (G″) moduli, with preserved structural integrity during the frequency-sweep testing [24]. F1 and F2 showed LVRs from 0.01% to 1.00% strain, and a strain of 0.10% was therefore selected for frequency-sweep measurements. The critical strain (γ_C_) was 1.00% for both gels. Beyond this point, a reduction in both G′ and G″ occurs, indicating the end of the LVR and a break in the gel structure [24]. Table 3 summarizes the LVR, applied strain within the LVR, and γC for F1 and F2 gels.

#### 2.4.3. Frequency-Sweep

**Figure 3:**
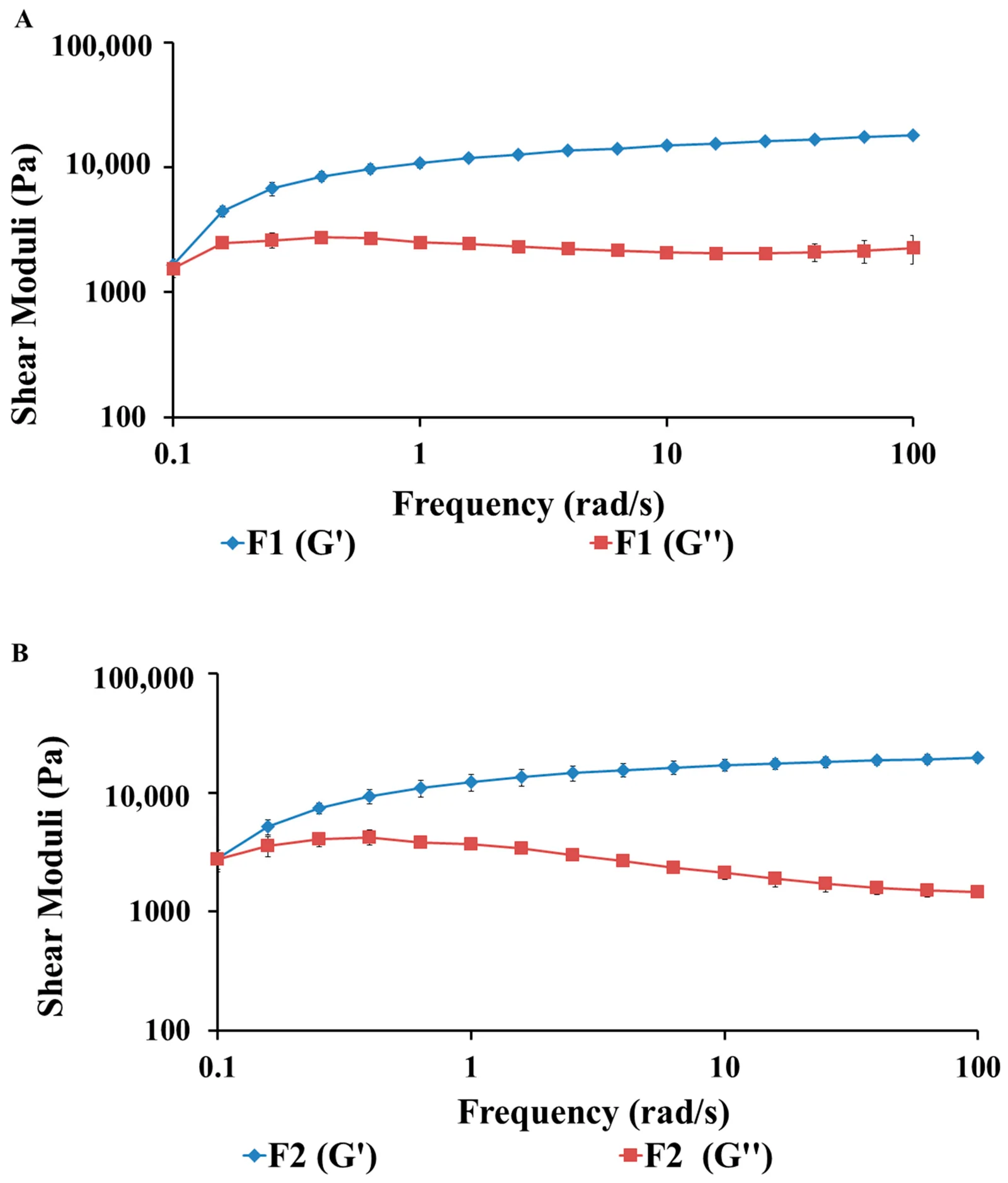
Frequency-dependent storage (G′) and loss (G″) moduli of (**A**) F1 and (**B**) F2 over 0.1–100 rad/s. Data are presented as mean ± SD (n = 3).

For both F1 and F2, the storage modulus (G′) dominated the loss modulus (G″) throughout the tested frequency range, indicating predominantly elastic behavior (Figure 3). F2 showed higher G′ and G″ values than F1, consistent with its higher viscosity.

#### 2.4.4. Temperature-Sweep

**Figure 4:**
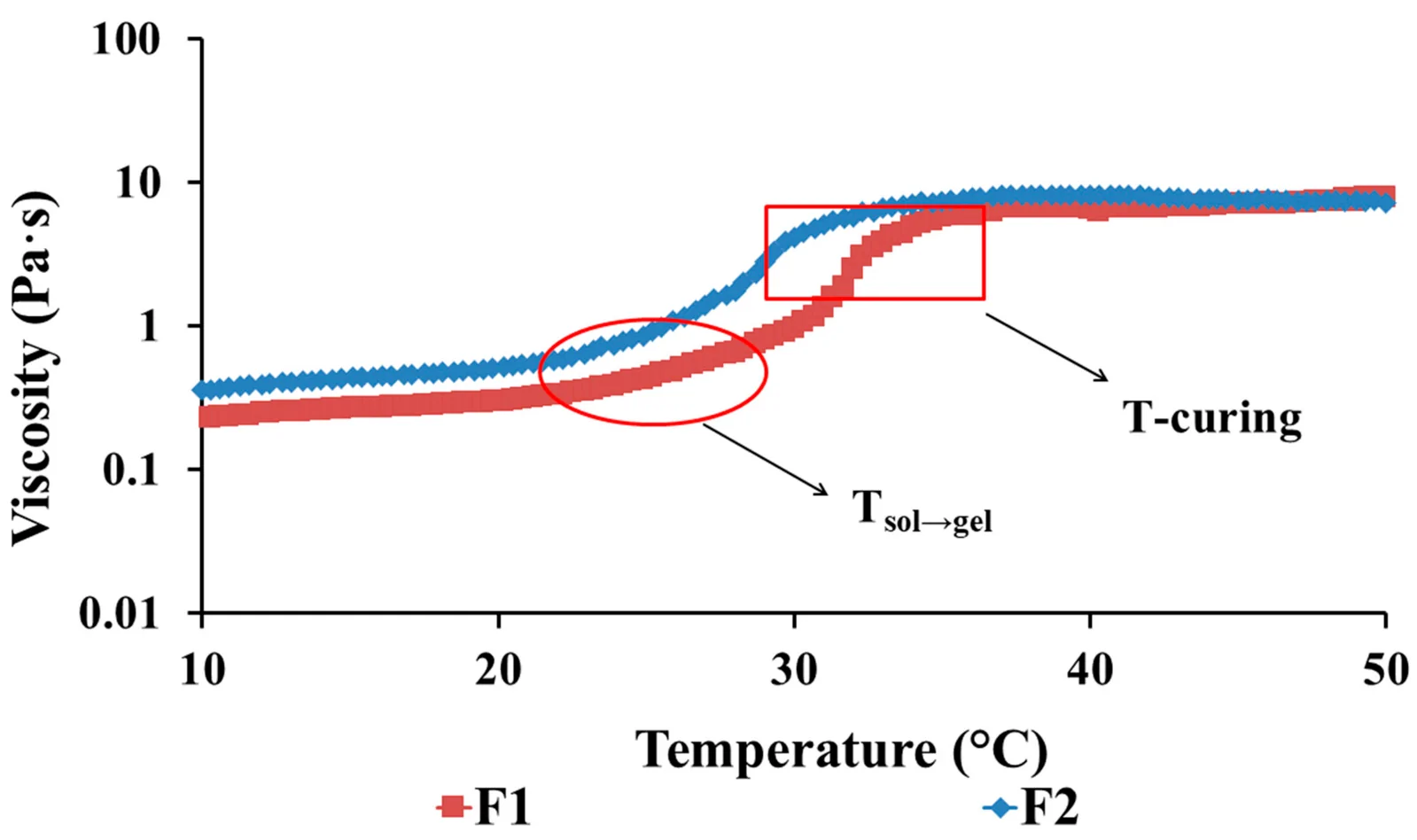
Temperature-sweep profiles showing the sol–gel transition and curing behavior of F1 and F2. Data are presented as mean ± SD (n = 3).

The temperature-sweep study showed a progressive increase in viscosity as F1 and F2 gels transitioned from sol to gel (Figure 4). Initially, viscosity was low at lower temperatures, indicating fluid systems and a sol phase. Viscosity then increased markedly with temperature due to micelle nucleation. Finally, viscosity plateaued, indicating complete gelation. The plateau viscosity was 100 times higher than that of the sol phase. The controlled stress rheometer (CSR)-derived Tsol → gel values were 28.26 ± 0.41 °C and 25.70 ± 0.20 °C for F1 and F2, respectively, whereas the corresponding curing temperatures were 34.54 ± 0.27 and 32.39 ± 0.79 °C. The Tsol → gel values obtained by the tube-inversion method of 33.70 ± 0.20 and 31.10 ± 0.45 °C for F1 and F2, respectively, were higher than the CSR rheometer-derived Tsol → gel values and close to the rheometer-derived curing temperatures.

The discrepancy between the Tsol → gel data obtained by the tube inversion method and those obtained by CSR is attributed to the different methods used. The tube-inversion method is considered the simplest approach for measuring Tsol → gel. The temperature at which no flow is observed when the tube is inverted is reported as the gelation temperature [26], thereby detecting the later macroscopic endpoint. While this method relies on visual observation and offers rapid determination of Tsol → gel with minimal equipment, it lacks precision and accuracy [27].

However, the CSR rheological methods are more reliable for investigating gel phase transitions [26]. The CSR is more sensitive to detecting the Tsol → gel at an early stage, before the formulation becomes non-flowing on a macroscopic scale. Therefore, the Tsol → gel values for F1 and F2 gels measured by CSR were lower than those measured by the tube inversion method. Rheological T-curing also supports this interpretation. T-curing represents a stage beyond the initial sol–gel transition, where the developing network is further strengthened toward a more fully developed gel structure. This difference between Tsol → gel and T-curing has also been reported for thermoresponsive hydrogels [24,28]. These findings align with Gioffredia et al. [29], who investigated the sol–gel transition of F127 solutions using temperature-sweep tests with a CSR at constant shear and different heating rates. Three characteristic temperatures were observed in the viscosity-temperature curve of 25% F127 gel: the gelation onset temperature at the viscosity of the sol phase, the inflection temperature at the curve flex, and the gelation temperature at the viscosity plateau [29].

#### 2.4.5. Rheological Stability Studies

The three-month stability assessment was limited to rheological properties of the extract-loaded gels (F1 and F2). Investigating the three-month rheological stability of the *P. brachyodon* extract-loaded in situ gels (F1 and F2) was essential because these gels are the final topical dosage forms used for wound healing. Therefore, assessing the viscosity and viscoelastic properties of these gels was essential to evaluate the structural integrity and ensure physical stability over time.

**Figure 5:**
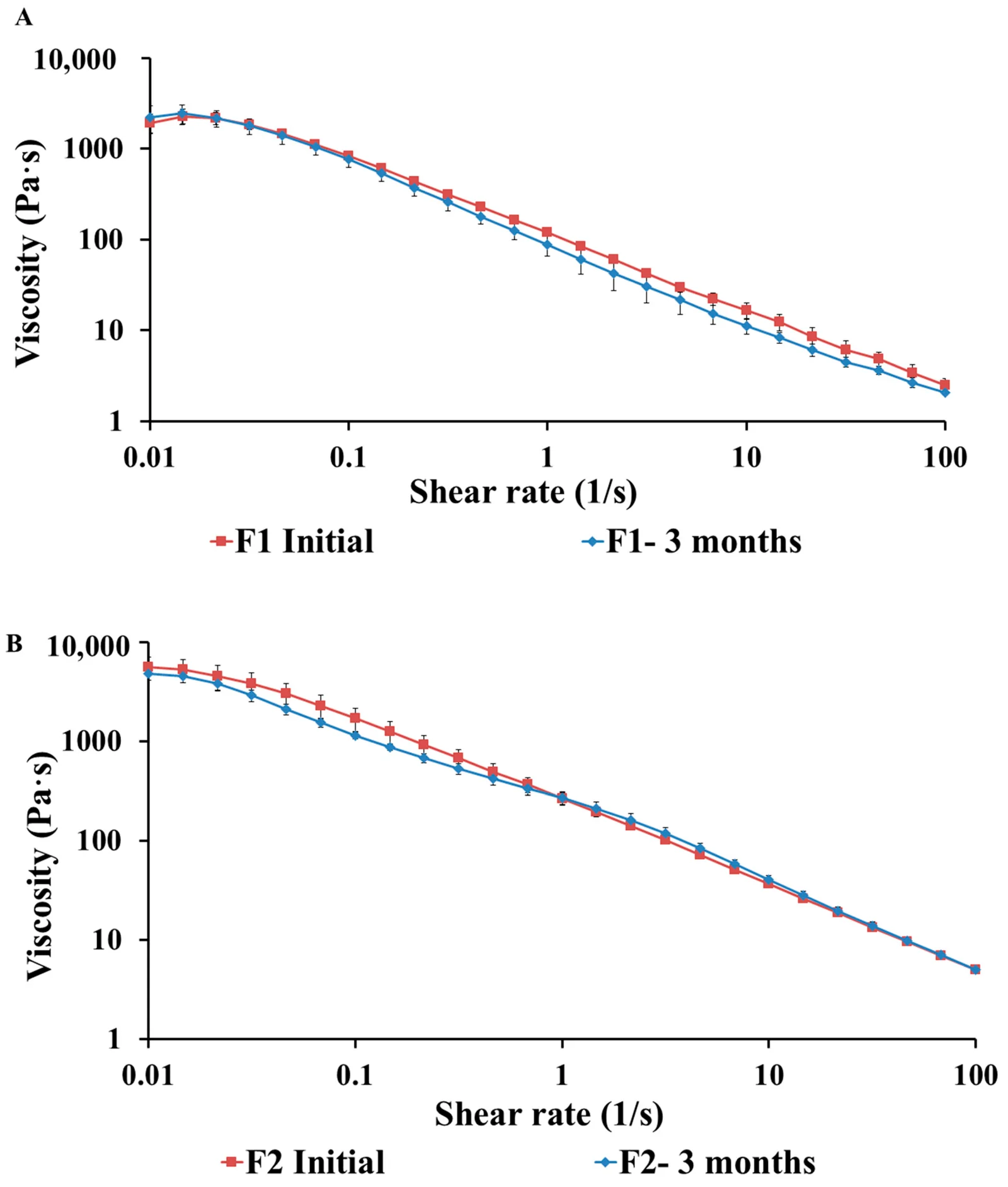
Viscosity profiles of (**A**) F1 and (**B**) F2 initially and after three months of storage at 4 °C. Data are presented as mean ± SD (n = 3).

**Figure 6:**
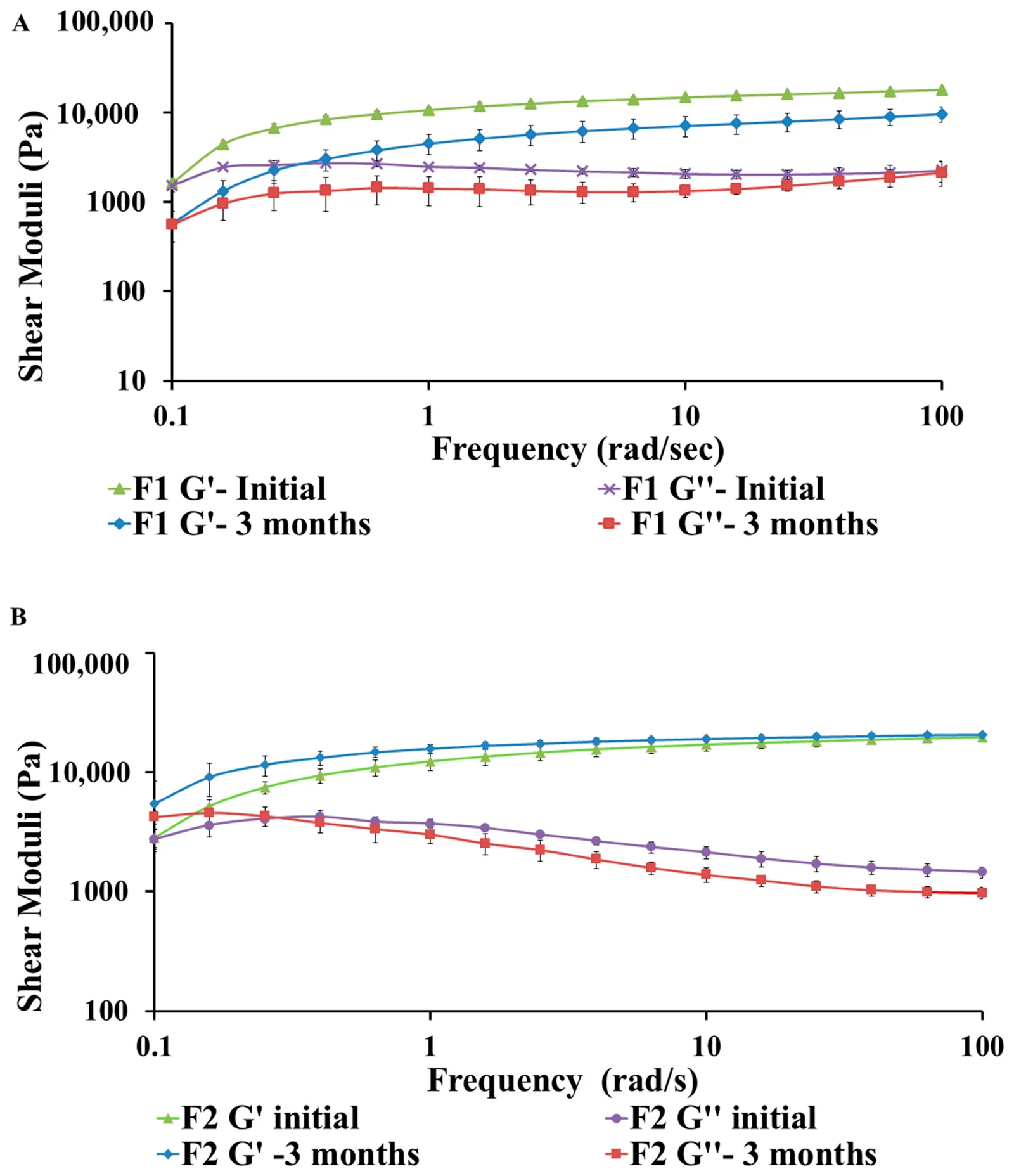
Frequency-dependent storage (G′) and loss (G″) moduli of (**A**) F1 and (**B**) F2 initially and after three months of storage at 4 °C. Data are presented as mean ± SD (n = 3).

After three months of storage at 4 °C, both gels retained shear-thinning behavior (Figure 5), and G′ remained greater than G″ (Figure 6). F1 showed a reduction in G′ and G″ during storage, whereas F2 showed smaller rheological changes.

### 2.5. Wound Healing Activity

**Figure 7:**
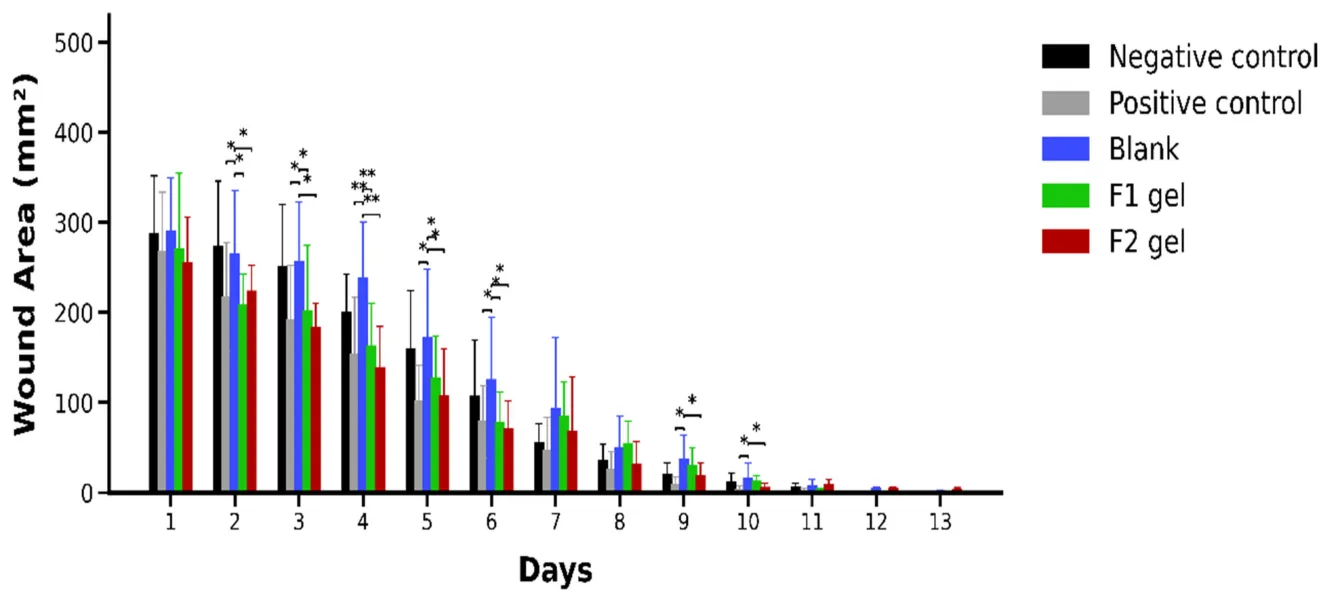
Wound areas in untreated (negative control), Herbaleen^®^ cream (positive control), G6 blank gel, F1, and F2 groups during the 15-day study. Data are presented as mean ± SD (n = 3). Significance symbols indicate the pairwise comparisons shown: * *p* < 0.05 and ** *p* < 0.01.

Linear mixed-effects analysis showed that wound-healing trajectories differed significantly among treatment groups over time (treatment-by-time trajectory: likelihood-ratio χ^2^(8) = 21.785, *p* = 0.0053). A progressive reduction in wound area was observed in all groups throughout the experimental period (Figure 7). However, the rate of wound closure differed among treatments, as demonstrated by the significant treatment-by-time trajectory in the linear mixed-effects analysis. This finding indicates that the effect of treatment was primarily reflected in the pattern of wound-area reduction over time rather than in a constant treatment effect across all assessment points.

Both extract-loaded formulations (F1 and F2) showed progressive wound-area reduction, with F2 generally showing a more pronounced reduction during the intermediate and late stages of healing compared with the blank formulation. F1 also showed accelerated wound-area reduction at several assessment points. The wound-level exploratory analysis provided additional descriptive support for differences between the formulations and G6 blank gel at selected time points. At Day 4, F2 differed from the G6 blank gel in the exploratory wound-level analysis after Holm adjustment (adjusted *p* = 0.0348).

F1 and F2 contained the same polymer types (F127 and F68) but at different concentrations; therefore, differences in wound healing cannot be attributed solely to extract type. The higher TPC, TFC, and DPPH radical-scavenging activity of the methanol extract may have contributed to the response observed with F2, but the contribution of formulation composition cannot be excluded. Moreover, the small group size and absence of quantitative mechanistic endpoints limit causal interpretation. The positive control (Herbaleen^®^ cream, Natural Echo, Amman, Jordan) showed progressive wound healing, whereas the G6 blank-gel and untreated groups generally showed slower closure and larger residual wound areas at intermediate assessment times.

**Figure 8:**
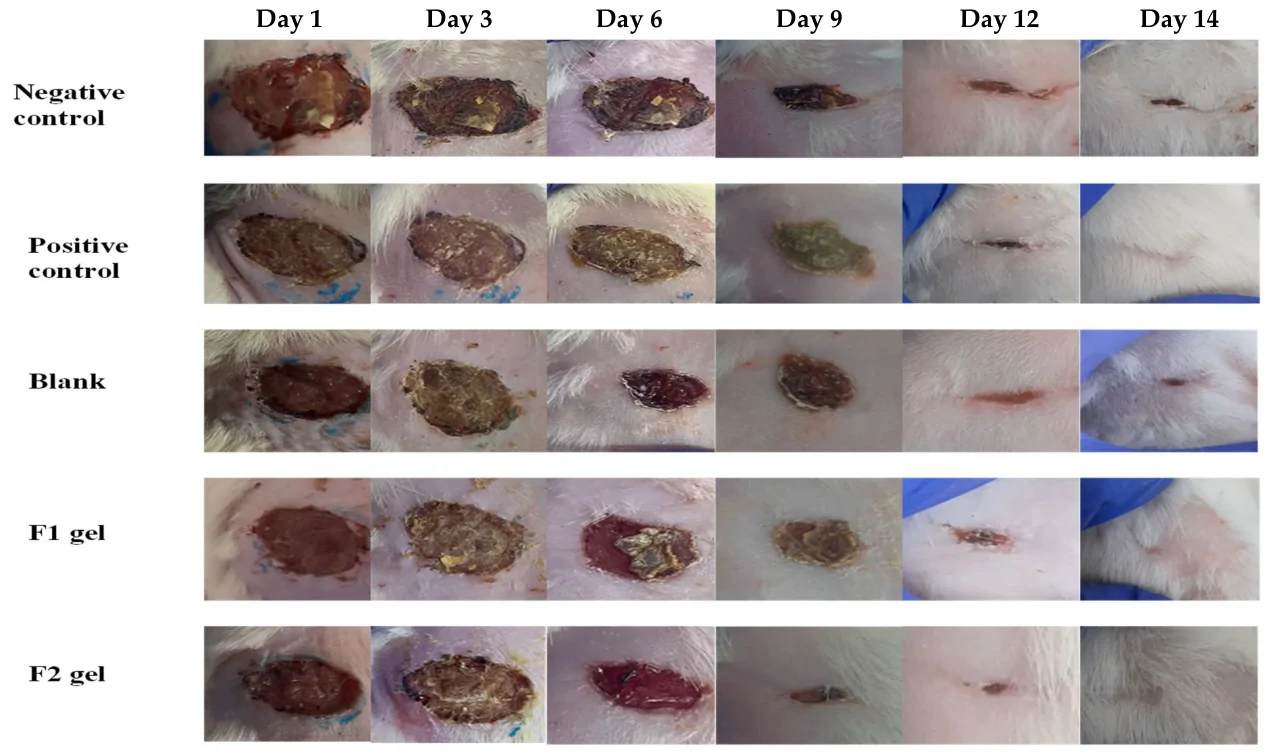
Representative wound photographs on days 1, 3, 6, 9, 12, and 14 in the untreated, positive-control (Herbaleen^®^ cream), G6 blank-gel, F1, and F2 groups.

Figure 8 presents representative macroscopic photographs of the wound healing progression across all groups over the 15-day observation period.

Previous studies on other *Phlomis* species support the wound-healing potential observed in the present study [30,31]. Okur et al. [30] demonstrated that the methanolic extract of *Phlomis russeliana* (*P. russeliana*), formulated as a topical gel, exerted in vivo wound-healing activity together with antioxidant and anti-inflammatory effects. The extract showed DPPH radical-scavenging activity (IC_50_ ≈ 0.91 mg/mL), while bioactivity-guided fractionation identified phytol and 1-heptadecanol among constituents associated with its biological activity. Similarly, the methanolic extract of *Phlomis rigida* (*P. rigida*) exhibited antioxidant activity in both DPPH and 2,2′-azino-bis(3-ethylbenzothiazoline-6-sulfonic acid) (ABTS) assays, lipoxygenase (LOX)-inhibitory activity (IC_50_ = 19.5 ± 2.8 µg/mL), and a dose-dependent enhancement of wound closure in vitro; hence, the incorporation of the extract into a topical gel also supported its wound-healing activity in vivo [31]. These studies [30,31] align with the present findings and support the potential contribution of antioxidant and anti-inflammatory phytochemicals of the genus *Phlomis* to wound repair.

More recent studies have emphasized the importance of the delivery system for improving the topical performance of *Phlomis* extracts [32,33]. Polymeric poly(lactic-co-glycolic acid) (PLGA) and PLGA-polyethylene glycol (PEG) nanoparticles containing *Phlomis crinite* (*P. crinite*) extract showed encapsulation efficiencies exceeding 90%, enhanced skin permeation of the major flavonoid constituent, and greater fibroblast migration and wound closure than the free extract [32]. Likewise, incorporating *P. crinita* extract into a nanostructured topical formulation resulted in sustained release and skin permeation of luteolin-acetylglucoside, and promoted migration and wound closure in fibroblast and keratinocyte models [33]. These findings support the rationale for incorporating *Phlomis*-derived phytochemicals into topical delivery systems rather than applying the crude extract alone. In the present study, the thermoresponsive F1 and F2 formulations similarly provide a structured topical vehicle for local delivery of *P. brachyodon* extracts.

### 2.6. Histology

**Figure 9:**
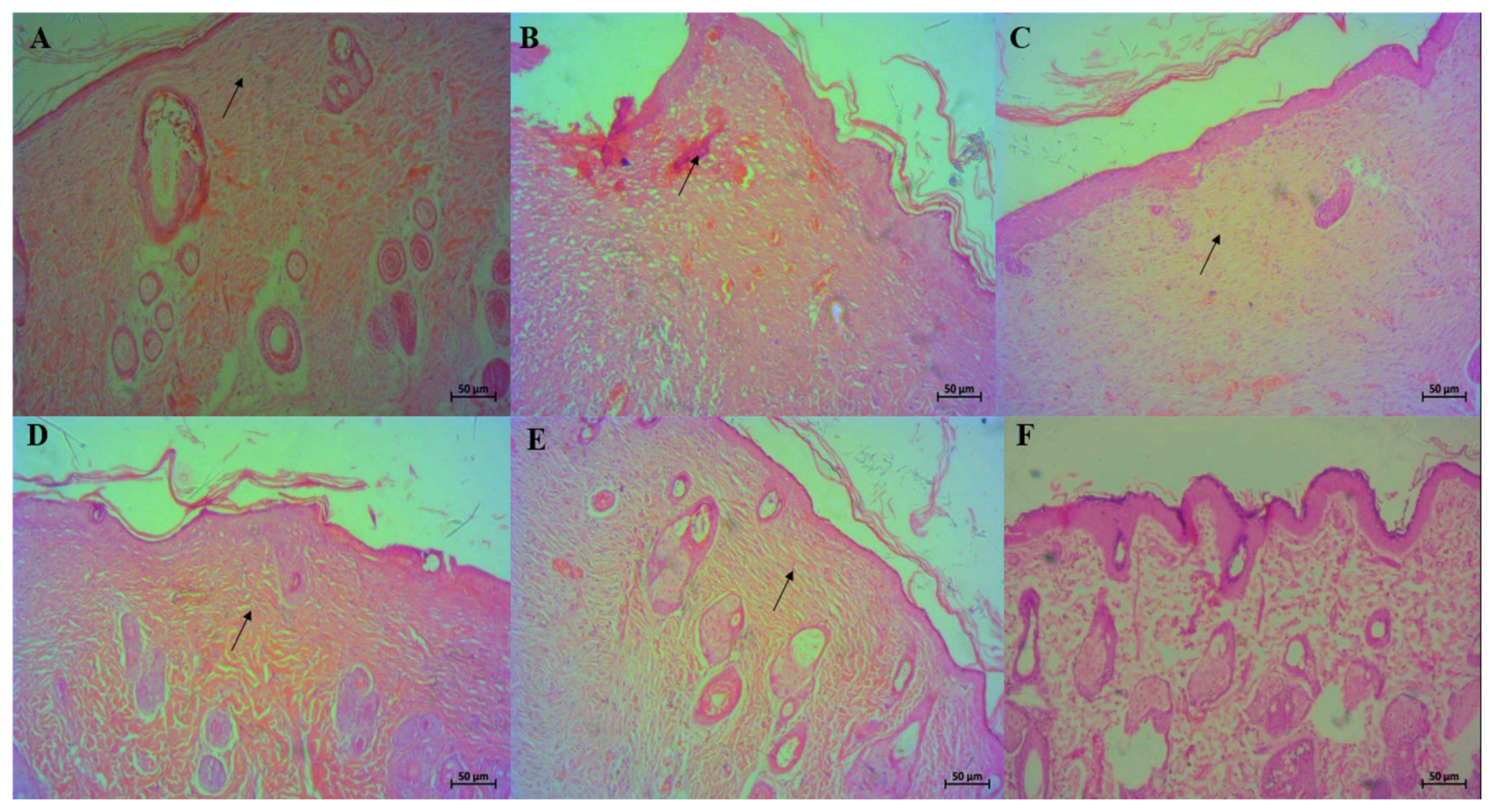
Representative H&E-stained wound sections on day 15: (**A**) untreated control, (**B**) positive control, (**C**) G6 blank gel, (**D**) F1, (**E**) F2, and (**F**) normal skin. Black arrows indicate representative areas of collagen deposition and organization within the dermis. Hematoxylin-stained nuclei appear blue-purple, whereas eosinophilic tissue components appear pink. Scale bars of 50 µm are shown in each panel.

Histological examination showed incomplete epidermal restoration and inflammatory cell infiltration in the untreated and blank-gel groups (Figure 9A,C). The F1 and F2 groups (Figure 9D,E) showed improved epidermal organization and reduced inflammatory infiltration relative to the untreated and blank-gel groups. The positive-control group (Figure 9B) showed advanced re-epithelialization and improved tissue organization, whereas normal skin (Figure 9F) showed intact architecture.

Histological examination was complemented with semiquantitative histopathological scoring and histomorphometric assessment (Table 4). Complete re-epithelialization was observed in all evaluated samples from the F1, F2, and positive-control groups, whereas incomplete re-epithelialization remained in individual untreated and G6 blank-gel samples. The positive-control group showed the most consistent keratinization, with no acute inflammatory infiltration and no residual granulation tissue. Both extract-loaded formulations showed similarly advanced reparative features. F2 showed complete re-epithelialization in all evaluated samples, no detectable acute inflammation, and descriptively greater collagen deposition, with increased neovascularization in some sections. F1 also showed complete re-epithelialization in all evaluated samples, although some inter-sample variability was observed in keratinization, granulation tissue, and acute inflammation. Chronic inflammatory scores were comparable across groups. Overall, the semiquantitative histological findings were consistent with the macroscopic wound-healing pattern and supported advanced tissue repair in the extract-loaded groups.

### 2.7. LC–MS Profile of P. brachyodon Extracts

LC–MS profiling detected 37 constituents in the butanol and methanol extracts, including phenolic acids, flavonoids, coumarins, and fatty acids (Table 5). The methanol extract showed greater chemical diversity, whereas a smaller number of high-intensity fatty-acid peaks dominated the butanol extract.

The butanol extract was dominated by palmitic acid (53.06% of the identified relative peak area) and 9-trans-palmitelaidic acid (37.24%). Chlorogenic acid, vitexin, quercetin, hyperoside, and acetoside were detected at lower relative peak areas. However, the methanol extract contained chlorogenic and ferulic acids, isoorientin, vitexin, daidzein, and kaempferol. The largest relative peak-area contributions included 9-trans-palmitelaidic acid (22.88%), naringenin (11.82%), α-linolenic acid (9.80%), pinolenic acid (9.51%), hispidulin (8.93%), apocynin (7.92%), and kumatakenin (4.65%). These percentages represent relative LC–MS peak areas.

### 2.8. Cell Viability Assays

Cell viability assays were performed for *P. brachyodon* butanol and methanol extracts using a normal fibroblast cell line (HGF) and an SCLC cell line (H69 cells). The HGF cell line, as a representative form of normal fibroblast cells, is commonly used to assess the possible cytotoxicity of extracts or novel compounds [34,35]. Table 6 presents the IC_50_ values for the extracts on HGF and H69 cell lines. HGF cells were used to assess the cytotoxicity of the extracts against normal cells and later to identify the selectivity of the extracts toward cancer cells, with the selectivity index (SI) values for the extracts also presented in Table 6.

The results showed that the butanol and methanol extracts of *P. brachyodon* are noncytotoxic on HGF cells at the highest concentration used (100 µg/mL) (Table 6). Neither the butanol nor methanol extract produced a 50% reduction in HGF cell viability at concentrations up to 100 µg/mL; therefore, HGF IC_50_ values were reported as >100 µg/mL. Hence, the cytotoxicity of the extracts on HGF cells may occur at much higher concentrations than those used in the formulations prepared in this study. Additionally, results obtained on HGF cells may help clarify the wound-healing effect achieved by the formulation, where the noncytotoxic concentration of the *P. brachyodon* extracts used may have facilitated cell proliferation and wound closure. This aligns with a previous study that showed an increase in cell viability in cells that were exposed to hydrogen peroxide (induced cytotoxicity) after treatment with Phlomis species extracts [36].

The methanol extract was more cytotoxic on H69 lung cancer cells than the butanol extract, with IC_50_ values of 2.24 µg/mL and 3.16 µg/mL, respectively (Table 6). These results align with a previous study that showed the potential anticancer activity of Phlomis species [18]. Furthermore, the SI values against H69 cells were lower-bound estimates (>31.65 for the butanol extract and >44.64 for the methanol extract) because the extracts’ IC_50_ values against HGF cells would exceed the highest tested concentration. Compounds or extracts with SI values above one are considered selective against cancer cells and may possess increased safety on normal cells [37,38]. However, tissue-matched normal lung cells are worth investigating to better identify the selectivity and safety of the extracts. These results provide insight into the potential anticancer activity of *P. brachyodon* extracts, which are worth investigating in other types of cancer as well.

## 3. Conclusions

The *P. brachyodon* extracts were successfully incorporated into a thermoresponsive gel base with suitable phase-transition and rheological characteristics. The methanol-extract formulation showed stronger antioxidant activity and the most rapid wound contraction in the rat excision model. These findings support further development of *P. brachyodon* topical formulations, but confirmation in adequately powered studies with quantitative histological and mechanistic endpoints is required. Future studies should focus on developing an analytical method to quantify the main component(s) of the *P. brachyodon* extract and on systematic MS/MS confirmation, reporting mass errors, and comparing them with authentic standards for major marker compounds. Additionally, future studies will focus on developing and validating marker-based analytical methods for the *P. brachyodon* extracts to enable quantitative assessment of content uniformity, in vitro release, skin permeation, and chemical stability of the gel formulations. Furthermore, *P. brachyodon* extracts have shown anticancer activity, particularly against H69 lung cancer cells; further testing on other cancer cell lines is warranted to validate the anticancer effect.

## 4. Materials and Methods

### 4.1. Plant Material

Aerial parts of *P. brachyodon* were collected in Jarash, Jordan, during the flowering period in April. The plant material was authenticated by one of the authors. Voucher specimens were deposited in the Department of Pharmaceutical Sciences, School of Pharmacy, The University of Jordan, and the Department of Pharmacy, Al-Zaytoonah (FMJ-LAMI-18-3).

### 4.2. Extraction

The aerial parts of *P. brachyodon* were air-dried, crushed, and macerated in 70% ethanol at room temperature for three weeks with occasional shaking. The resulting extract was filtered and concentrated under reduced pressure at 40 °C using a rotary evaporator, yielding the crude ethanol extract. This extract was redissolved in 50% aqueous methanol and defatted with *n*-hexane in a separatory funnel to remove chlorophylls and other lipophilic substances. The remaining aqueous methanol layer was fractionated by polarity-based liquid–liquid extraction. *n*-Butanol was used to separate intermediate-polarity phytochemicals, while highly polar compounds remained in the aqueous-methanol fraction. The resulting fractions (hexane-free methanol extract, butanol fraction, and aqueous fraction) were collected, dried, and stored at −20 °C until further analysis.

### 4.3. Liquid Chromatography Mass Spectrometry (LC-MS)

LC–MS analysis of the *P. brachyodon* butanol and methanol extracts was performed using a Bruker Impact II ESI-Q-TOF mass spectrometer coupled to a Bruker Elute UPLC system and an Apollo II ion-funnel electrospray source (Bruker Daltonics GmbH & Co. KG, Bremen, Germany). Samples were dissolved in DMSO, diluted with acetonitrile, centrifuged at 4000 rpm for 2 min, and 3 µL was injected. Separation was achieved using an Intensity Solo C18 UHPLC column (100 × 2.1 mm, 2.0 µm; Bruker Daltonics GmbH & Co. KG, Bremen, Germany) at 40 °C and 0.51 mL/min using water containing 0.05% formic acid (A) and acetonitrile (B): 5–80% B over 0–27 min, 95% B at 27–29 min, and return to 5% B at 29.1 min; total run time was 35 min. Analyses were performed in positive and negative ionization modes. Compounds were annotated using accurate mass, retention behavior, and comparison with available authentic standards and published spectral data [39].

### 4.4. Total Phenol Content (TPC)

Total phenolic content of the *P. brachyodon* butanol and methanol extracts was determined using the Folin–Ciocalteu method. A five-point calibration curve (6.125–100 µg/mL) was prepared using gallic acid, and methanol served as the blank. Results were expressed as milligrams of gallic acid equivalents per gram of extract (mg GAE/g) [40].

### 4.5. Total Flavonoid Content (TFC)

Total flavonoid content of the *P. brachyodon* butanol and methanol extracts was determined using the aluminum chloride colorimetric method [41]. Methanol was used as the blank. TFC values were expressed as mg of rutin equivalents per gram of extract (mg RE/g).

### 4.6. Antioxidant Activity

Antioxidant activity of the *P. brachyodon* butanol and methanol extracts was evaluated using the DPPH radical-scavenging assay. The IC_50_ value was defined as the extract concentration required to scavenge 50% of DPPH radicals [42].

### 4.7. Preparation of Blank Thermoresponsive In Situ Gels

Blank thermoresponsive in situ gels (G1–G7) were prepared using the cold method with different concentrations of Pluronic F127 (F127) and Kolliphor P188 (F68) [24]. The polymers were dispersed in cold ultrapure water (UPW) in an ice bath and stirred continuously for 6 h. The formulations were then stored at 2–8 °C for 24 h to ensure complete polymer hydration and dissolution. Table 7 illustrates the composition of the blank thermoresponsive in situ gels (G1–G7).

### 4.8. Preparation of P. brachyodon Extract-Loaded In Situ Gels

A 2% (*w*/*v*) stock solution of each *P. brachyodon* extract (butanol and methanol) was prepared by dissolving 200 mg of extract in 0.7 mL of ethanol and adjusting the volume to 10 mL. To prepare F1, 7.2 mL of the 2% butanol-extract stock solution (144 mg extract) was incorporated into 10 g of G6 blank gel under gentle stirring in an ice bath. To prepare F2, 8.2 mL of the 2% methanol-extract stock solution (164 mg extract) was incorporated into 12 g of G7 blank gel under gentle stirring in an ice bath. The butanol extract-loaded gel was designated F1 and the methanol extract-loaded gel F2. The prepared gels were stored at 4 °C for 24 h before evaluation.

### 4.9. Characterization of P. brachyodon Extract-Loaded In Situ Gels

#### 4.9.1. Phase Transition Behavior

The sol–gel transition temperature (Tsol → gel) and gelation time (Tgel) of *P. brachyodon* extract-loaded in situ gels (F1 and F2) were determined using the tube inversion method as described in [24]. Samples were heated from 20 to 50 °C in a water bath. Tsol → gel and Tgel were recorded when the formulation no longer flowed upon inversion.

#### 4.9.2. Rheological Measurements

A controlled-stress rheometer (CSR) (Anton Paar MCR 302, Graz, Austria) was utilized to evaluate the rheological properties of the *P. brachyodon* extract-loaded in situ gels (F1 and F2), using a cone/plate configuration with a 1º cone angle and a 25 mm plate diameter.

##### Viscosity

The viscosity of F1 and F2 gels was measured at 32 °C as described in [24]. A 0.5 g sample of each gel was placed onto the CSR. After a 1 min equilibration period, viscosity was recorded at shear rates of 0.01–100 s^−1^. Measurements were performed in triplicate.

##### Amplitude-Sweep

To determine the linear viscoelastic region (LVR) of F1 and F2 gels, dynamic oscillatory shear testing was conducted at 32 °C using the CSR, as outlined by [24]. A 0.5 g sample of each gel was placed on the plate and left to rest for about 1 min. The oscillation frequency was set to 6.28 rad/s during the study. The LVR of the gels was evaluated at strain levels from 0.01% to 100%. The LVR for each gel was assessed in triplicate.

##### Frequency-Sweep

The viscoelastic properties of the in situ gels F1 and F2 were evaluated as described in [24]. The gels were subject to frequency-sweep analysis using the CSR. All measurements were performed over the frequency range of 0.1 to 100 rad/s at a temperature of 32 °C. Constant strain within the LVR was determined based on the amplitude-sweep analyses of each gel. A 0.5 g sample of each gel was placed on the plate and allowed to rest for about 1 min. The frequency-dependent elastic modulus (G′) and viscous modulus (G″) were assessed by lowering and oscillating the top cone. Each gel was assessed in triplicate.

##### Temperature-Sweep

A controlled-stress rheometer was employed to determine the Tsol → gel and curing temperatures (T-curing) of the in situ gels F1 and F2 as described in [24]. Temperature-sweep measurements were conducted at a heating rate of 5 °C/min and a constant shear rate of 50 s^−^^1^ over a temperature range of 10–50 °C. Each gel was evaluated in triplicate.

##### Rheological Stability Studies

The rheological stability of F1 and F2, including viscosity and viscoelastic properties, was evaluated after three months of storage at 4 °C using the CSR. The stability results of F1 and F2 were compared to the initial findings to identify any changes over time.

### 4.10. In Vivo Wound Healing Activity of P. brachyodon Extract-Loaded In Situ Gels

#### 4.10.1. Animals

Female albino rats (n = 15; age, 8 weeks; body weight between 180 and 220 g) were housed under controlled conditions (27 ± 1 °C) with free access to standard feed and water. Bedding was replaced twice weekly. All animal procedures were approved by the Institutional Animal Care and Use Committee of Al-Zaytoonah University of Jordan (IRB no. 30/12/2025-2026).

#### 4.10.2. Excision Wound Formation

This pilot wound-healing study used female albino rats in the Experimental Animal House at Al-Zaytoonah University of Jordan and followed an excision-wound model [43]. Hair on the dorsal surface was shaved one day before wound induction. Under general anesthesia and aseptic conditions, two full-thickness circular excision wounds (2 cm diameter) were created on the dorsal skin, one on each side. Animals were anesthetized by inhalation of diethyl ether using a desiccator until an adequate surgical plane of anesthesia was achieved. Each rat therefore contributed two wound measurements at each assessment time point; for the primary longitudinal analysis, the two measurements were averaged within rat so that the animal, rather than the individual wound, served as the independent experimental unit (n = 3 animals per group).

After recovery, animals were housed individually and received the assigned topical treatment for 15 days. The 15 rats were randomly allocated to five groups (n = 3 per group): Group 1 was treated with butanol extract-loaded in situ gel (F1), Group 2 was treated with methanol extract-loaded in situ gel (F2), Group 3 was treated with G6 blank gel composed of 19.0% F127 and 9.0% F68, Group 4 was the untreated negative control, and Group 5 was treated with Herbaleen^®^ cream (Natural Echo, Amman, Jordan), used as a positive control.

#### 4.10.3. Treatment Administration and Wound-Area Assessment

Beginning one day after wound induction, the entire wound area was treated once daily for 15 days with 800 µL of F1, F2, or G6 blank gel. The positive-control group received Herbaleen^®^ cream, and the negative-control group received no treatment. F1 and F2 were prepared from G6 and G7, respectively. Both vehicles contained F127 and F68, but at different concentrations (G6: 19.0% F127 and 9.0% F68; G7: 20.8% F127 and 10.0% F68; Table 7). Because a matched G7 blank-control group was not included, formulation-composition effects cannot be separated completely from extract effects; therefore, comparisons involving F2 are interpreted with this limitation in mind.

Standardized wound photographs were obtained at the scheduled assessment time points, and wound area was quantified from the images using ImageJ software, version 2 (National Institutes of Health, Bethesda, MD, USA). The resulting measurements were used for the longitudinal statistical analysis.

### 4.11. Histology

Tissue samples collected on day 15 were fixed in neutral buffered formalin, processed through graded ethanol, cleared in xylene, embedded in paraffin, and sectioned at 4–5 µm. Sections were stained with hematoxylin and eosin and examined under a light microscope [44].

Histological assessment was performed on tissue samples collected on day 15 from three animals per experimental group (n = 3/group). H&E-stained sections were coded and evaluated in a blinded manner with respect to treatment allocation using a semiquantitative wound-healing scoring system as described in [45,46,47,48] with a slight modification. The evaluated parameters included re-epithelialization, keratinization, epithelial/epidermal thickness, epidermal thickness index (ETI), granulation tissue, granulation tissue/fibroblast maturation, tissue remodeling, scar elevation index (SEI), acute and chronic inflammation, neovascularization, and collagen deposition. Epithelial/epidermal thickness was measured in millimeters (mm) at multiple points per section. Parameters were evaluated according to predefined histological criteria and assigned ordinal scores where applicable. The semiquantitative histological scores were interpreted as supportive measures of tissue repair in this pilot study.

ETI and SEI were calculated as wound-area-to-uninjured-skin thickness indices. ETI was calculated using the following equation:

$$ETI=\frac{P2}{P1}\;\times 100$$

where P2 is the average epidermal thickness in the wound area, and P1 is the average epidermal thickness in uninjured skin. SEI was calculated analogously using average dermal thickness. In the scoring worksheets, ratios such as 2/1 and 1/1 denote the P2/P1 thickness ratio before conversion to a percentage (e.g., 2/1 = 200% and 1/1 = 100%). For both ETI and SEI, values of 95–105% were considered normal (score 2), values > 105% indicated hypertrophy (score 1), and values < 95% indicated hypoplasia (score 0).

### 4.12. Cell Viability Assays

#### 4.12.1. Cell Subculture and Growth Conditions

Cell culture media (DMEM and RPMI-1640) and phosphate-buffered saline (PBS) were purchased from Euroclone (Pero, Italy). Fetal Bovine Serum (FBS) was purchased from Capricorn Scientific, Ebsdorfergrund, Germany. Human gingival fibroblast cell line (HGF; ATCC code: PCS-201-018) and small-cell lung cancer (SCLC) cell line (H69; ATCC code: HTB-119) were purchased from ATCC, VA, USA. HGF cells were grown in 10% (*v*/*v*) FBS/DMEM (complete medium). H69 cells were grown in 10% (*v*/*v*) FBS/RPMI (complete medium). Cells were incubated in a humidified 5% CO_2_ atmosphere at 37 °C. HGF cells were grown as adherent cells, whereas the H69 cell line was grown as suspended cells.

#### 4.12.2. Cell Viability Assays Using the Resazurin Dye Method

Resazurin sodium salt and dimethyl sulfoxide (DMSO) were purchased from Sigma-Aldrich (St. Louis, MO, USA). The resazurin dye colorimetric method was used to assess cell viability, as described in [49]. The seeding densities for HGF and H69 cells were 2500 cells/200 µL/well and 1 × 10^4^ cells/180 μL/well, respectively, all seeded in 96-well plates. For the adherent cells, after 24 h of incubation, 180 µL of fresh complete medium was added to each well following the removal of the old medium. Stock solutions of *P. brachyodon* butanol and methanol extracts (10 mg/mL each) were prepared by dissolving them in DMSO, then serially diluted in complete medium of the respective cell line to be used, forming several diluted stock solutions ranging from 1000 to 0.1 µg/mL. Then, 20 μL of the extract-diluted stock solutions were added for 72 h of treatment. The final well concentrations of the extracts ranged from 100 µg/mL to 0.01 µg/mL. Wells serving as untreated controls and for background fluorescence were prepared. After 72 h, resazurin dye (20 μL; 0.125 mg/mL in PBS) was added to each well, and plates were incubated for 2 h (HGF cells) or for 4 h (H69 cells). Fluorescence readings (excitation 540 nm/emission 620 nm) were recorded using a BioTeK SYNERGY HTXmulti-mode plate reader (BioTek Instruments, Winooski, VT, USA), and percentage viability was calculated relative to the control (untreated) cells. Data are presented as means and standard error of the mean (SEM). The IC_50_ values were calculated using nonlinear regression analysis. GraphPad Prism version 9.3 was used to carry out the analysis.

### 4.13. Statistical Analysis

Data are presented as mean ± standard deviation (SD) unless otherwise stated. For wound healing, the two wound-area measurements from each rat were averaged at each assessment time point, and the rat was treated as the independent experimental unit. Longitudinal wound-area data were analyzed in IBM SPSS Statistics, version 27 (IBM Corp., Armonk, NY, USA) using a linear mixed-effects model with treatment group, time, quadratic time, and the treatment-by-time terms as fixed effects and animal as a random effect to account for repeated measurements within rats. Model comparisons were evaluated using likelihood-ratio testing, and a two-sided *p*-value < 0.05 was considered statistically significant. Exploratory wound-level comparisons between treated groups and the G6 blank-gel group at individual time points were performed using Welch’s t-test with Holm adjustment for multiple comparisons; because two wounds originated from each rat, these wound-level comparisons were considered exploratory rather than independent confirmatory analyses. Graphs were generated using GraphPad Prism version 9.3.

For histological assessment, the histopathological ordinal scores were summarized as the median and range. Given the pilot nature of the study and the small group size (n = 3 animals/group), between-group histological comparisons were considered exploratory. Continuous epithelial/epidermal thickness measurements, obtained in mm from multiple points per section, were summarized descriptively as mean ± SD. Where appropriate, ordinal histological parameters were analyzed using the Kruskal–Wallis test. A two-sided *p*-value < 0.05 was considered statistically significant.

## Figures and Tables

**Table 1:** Total phenol content (TPC) and total flavonoid content (TFC), and IC_50_ values of *P. brachyodon* extracts in the DPPH assay. Data are expressed as mean ± SD (n = 3).

Extract	TPC (mg GAE/g)	TFC (mg RE/g)	IC_50_ (µg/mL)
Butanol	94.29 ± 3.92	446.31 ± 24.73	144.11 ± 10.95
Methanol	183.96 ± 8.44	1115.64 ± 42.09	55.82 ± 8.94
Ascorbic acid	-	-	6.96 ± 3.53

**Table 2:** Appearance and phase-transition characteristics of *P. brachyodon* extract-loaded thermoresponsive gels.

*P. brachyodon* Extract-Loaded In Situ Gels	Clarity	Tsol → Gel (°C)	Tgel (min)	Appearance
F1	Clear	33.7 ± 0.2	3.70 ± 0.10	Dark yellow
F2	Turbid	31.1 ± 0.45	3.20 ± 0.09	Dark green

**Table 3:** Rheological parameters of the amplitude-sweep tests of F1 and F2 gels (LVR, applied strain within LVR, and critical strain (γ_C_)).

*P. brachyodon* Extract-Loaded In Situ Gels	LVR (%)	Applied Strain Within the LVR (%)	Critical Strain (γ_C,_ %)
F1	0.01–1.00	0.10	1.00
F2	0.01–1.00	0.10	1.00

**Table 4:** Semiquantitative histopathological assessment of wound healing on day 15.

Parameter	Untreated	G6 Blank	F1	F2	Positive Control
Epithelial/epidermal thickness (mm), mean ± SD	0.40 ± 0.20	0.50 ± 0.10	0.48 ± 0.16	0.37 ± 0.06	0.47 ± 0.15
Re-epithelialization	2 (1–2)	2 (1–2)	2 (2–2)	2 (2–2)	2 (2–2)
Keratinization	1 (0–2)	1 (0–2)	2 (1–2)	1 (1–2)	2 (2–2)
Granulation tissue	0 (0–1)	0 (0–1)	0 (0–1)	0 (0–0)	0 (0–0)
Granulation tissue/fibroblasts	1 (1–2)	1 (1–1)	1 (1–1)	1 (1–2)	1 (1–1)
Remodeling	1 (1–2)	1 (1–1)	1 (1–1)	1 (1–1)	1 (1–2)
Acute inflammation	0 (0–1)	0 (0–1)	0 (0–1)	0 (0–0)	0 (0–0)
Chronic inflammation	1 (1–1)	1 (1–1)	1 (1–1)	1 (1–1)	1 (1–1)
Neovascularization	1 (1–1)	1 (1–1)	1 (1–1)	1 (1–2)	1 (1–1)
Collagen deposition	1 (1–1)	1 (1–1)	1 (1–1)	2 (1–2)	1 (1–1)

SD: standard deviation; Scores are presented in the form of [Median (Range)].

**Table 5:** LC–MS profile of the butanol and methanol extracts of *P. brachyodon*. RT, retention time; M meas, measured mass; AUC, relative area under the chromatographic peak.

					Butanol Extract		Methanol Extract	
	RT [min]	M Meas.	Name	Molecular Formula	AUC	% Within Identified Compounds	AUC	% Within Identified Compounds
1	1.50	138.0	Protocatechuic aldehyde	C_7_H_6_O_3_	-	-	4772	0.53
2	2.63	267.1	Adenosine	C_10_H_13_N_5_O_4_	-	-	13,264	1.46
3	2.71	152.0	Vanillin	C_8_H_8_O_3_	86	0.21	-	-
4	3.89	354.1	Chlorogenic acid	C_16_H_18_O_9_	740	1.79	24,240	2.67
5	3.89	162.0	4-OH-coumarin	C_9_H_6_O_3_	536	1.29	33,384	3.68
6	4.45	198.1	Gallic acid ethyl ester	C_9_H_10_O_5_	-	-	1560	0.17
7	4.91	192.0	Scopoletin	C_10_H_8_O_4_	-	-	1486	0.16
8	5.69	122.0	Benzoic acid	C_7_H_6_O_2_	-	-	1524	0.17
9	6.02	166.2	Apocynin	C_9_H_10_O_3_	-	-	71,858	7.92
10	6.27	178.0	Aesculetin	C_9_H_6_O_4_	-	-	8284	0.91
11	7.19	432.1	Vitexin	C_21_H_20_O_10_	780	1.88	3210	0.35
12	7.72	610.2	*Neo*-hesperidin	C_28_H_34_O_15_	128	0.31	-	-
13	8.82	254.1	Daidzein	C_15_H_10_O_4_			10,794	1.19
14	9.10	302.0	Quercetin	C_15_H_10_O_7_	488	1.18	-	-
15	9.20	464.1	Hyperoside	C_21_H_20_O_12_	308	0.74	-	-
16	9.89	448.1	Isosakuranin	C_22_H_24_O_10_	-	-	17,126	1.89
17	9.94	446.1	Sissotrin	C_22_H_22_O_10_	-	-	1336	0.15
18	10.05	448.1	Isoorientin	C_21_H_20_O_11_	-	-	20,622	2.27
19	10.30	610.2	Hesperidin	C_28_H_34_O_15_	-	-	1876	0.21
20	11.03	624.2	Acteoside	C_29_H_36_O_15_	174	0.42	8694	0.96
21	11.05	194.1	Ferulic acid	C_10_H_10_O_4_	-	-	18,056	1.99
22	11.60	300.1	5,7-Dimethoxy-4′-OH-flavanone	C_17_H_16_O_5_	-	-	4318	0.48
23	14.96	272.1	Naringenin	C_15_H_12_O_5_	-	-	107,194	11.82
24	15.82	286.0	Kaempferol	C_15_H_10_O_6_	-	-	5220	0.58
25	15.92	300.1	Ladanetin	C_16_H_12_O_6_	158	0.38	-	-
26	17.47	110.0	Catechol	C_6_H_6_O_2_	-	-	9750	1.07
27	17.70	300.1	Hispidulin	C_16_H_12_O_6_	-	-	81,008	8.93
28	17.94	330.1	7,3′-dimethoxy-5,6,4′-tri-OH-isoflavone	C_17_H_14_O_7_	-	-	23,852	2.63
29	19.03	502.3	Medicagenic acid	C_30_H_46_O_6_	-	-	2390	0.26
30	19.05	314.1	Kumatakenin	C_17_H_14_O_6_	-	-	42,150	4.65
31	26.78	298.2	3-*trans*-OH-octadec-7-enoic acid	C_18_H_34_O_3_	620	1.50	-	-
32	26.96	278.2	a-Linolenic acid	C_18_H_30_O_2_	-	-	88,892	9.8
33	27.97	278.2	Pinolenic acid	C_18_H_30_O_2_	-	-	86,306	9.51
34	29.46	254.2	9-*trans*-Palmitelaidic acid	C_16_H_30_O_2_	15,432	37.24	207,564	22.88
35	29.73	414.3	Lupulone	C_26_H_38_O_4_	-	-	1194	0.13
36	29.84	256.2	Palmitic acid	C_16_H_32_O_2_	21,984	53.06	-	-
37	30.02	242.2	Pentadecanoic acid	C_15_H_30_O_2_	-	-	5272	0.58

**Table 6:** IC_50_ values (µg/mL ± SEM) of the extracts on HGF and H69 cell lines after 72 h treatment; n = 3. SI values for the extracts are also presented.

*P. brachyodon* Extracts	HGF	H69	SI Value for H69
	IC_50_ ± SEM (µg/mL)	IC_50_ ± SEM (µg/mL)	
Butanol extract	>100	3.16 ± 0.08	>31.65
Methanol extract	>100	2.24 ± 0.20	>44.64

**Table 7:** The composition of the blank thermoresponsive in situ gels (G1–G7).

Blank Gels	F127 (g, % *w*/*v*)	F68 (g, % *w*/*v*)	q.s. UPW (g, mL)
G1	2.0, 12.5%	1.0, 6.3%	16.0
G2	2.0, 14.3%	1.0, 7.1%	14.0
G3	2.0, 16.7%	1.0, 8.3%	12.0
G4	2.0, 20.0%	1.0, 10.0%	10.0
G5	2.0, 18.2%	1.0, 9.1%	11.0
G6	1.9, 19.0%	0.9, 9.0%	10.0
G7	2.5, 20.8%	1.2, 10.0%	12.0

## Data Availability

The data presented in this study are openly available in the article.

## Abbreviations

AUC	Area under the peak
CSR	Controlled-stress rheometer
DPPH	2,2-diphenyl-1-picrylhydrazyl
F127	Pluronic F127
F68	Kolliphor P188
G′	Storage modulus
G″	Loss modulus
H&E	Hematoxylin and eosin
IC_50_	Half-maximal inhibitory concentration
LC–MS	Liquid chromatography–mass spectrometry
LVR	Linear viscoelastic region
TFC	Total flavonoid content
TPC	Total phenolic content
